# Family planning, sexual activity and contraception in hereditary hemorrhagic telangiectasia: a European survey study

**DOI:** 10.1186/s13023-025-03887-x

**Published:** 2025-08-01

**Authors:** Josefien Hessels, Marco C. Post, Sanne Boerman, Freya Droege, Olivier Dupuis, ePAG, Claudia Crocione, Christina Grabowski, Ria Blom, Luisa M. Botella, Fernando Brocca, Didier Erasme, Paolo Federici, Mildred Lundgren, Tone Soderman, Karen T. Druckman, Dara Woods, Urban W. Geisthoff, Pernille D. Haahr, Anette D. Kjeldsen, Johannes-Jurgen Mager, Sophie Dupuis-Girod, Elisabetta Buscarini

**Affiliations:** 1 Pulmonology Department, St. Antonius Hospital, Nieuwegein, The Netherlands; 2 VASCERN HHT European Reference Centre and Department of Cardiology, St. Antonius Hospital, Nieuwegein, The Netherlands; 3 Division of Heart and Lungs, University Medical Centre Utrecht, Utrecht, The Netherlands; 4 VASCERN HHT European Reference Centre and Otorhinolaryngology Department, Essen University Hospital, Essen, Germany; 5 Department of Obstetrics and Gynecology, Hospices Civils de Lyon, Lyon, France; 6 VASCERN HHT European Reference Centre and Department of Otorhinolaryngology, Head and Neck Surgey, University Hospital of Marburg and Phillips Universität Marburg, Marburg, Germany; 7 VASCERN HHT European Reference Centre and Department of Otorhinolaryngology Head and Neck Surgery, Odense University Hospital, Odense, Denmark; 8 VASCERN HHT European Reference Centre OUH and Department of Otorhinolaryngology Head and Neck Surgery, Odense University Hospital, Odense, Denmark; 9 VASCERN HHT European Reference Centre and Department of Pulmonology, St. Antonius Hospital, Nieuwegein, The Netherlands; 10 VASCERN HHT European Reference Centre and Hospices Civils de Lyon, HHT National Reference Center and Genetic Department, Hôpital Femme-Mère-Enfants, Bron, France; 11 VASCERN HHT European Reference Centre and Department of Gastroenterology, ASST Ospedale Maggiore Crema, Crema, Italy

**Keywords:** Hereditary hemorrhagic telangiectasia, Family planning, Sexual activity, Intimacy, Contraception, Questionnaire

## Abstract

**Background:**

Hereditary hemorrhagic telangiectasia (HHT) can influence the quality of life and social relationships, mostly due to epistaxis, but the topics of family planning, sexual activity and contraception have not been investigated to date. This study aimed to gain more insight: what is the influence of HHT on family planning, sexual activity and contraception?

**Methods:**

This multi-language European survey study included a patient’s and partners’ version of a questionnaire designed specifically for this study. HHT patients were informed about the study through HHT expert centres, social media and websites of patient associations. Data collection took place between March- May 2023.

**Results:**

The survey was completed by 572 patients with a definite HHT diagnosis, based on genetic confirmation or ≥ 3 Curaçao criteria. In most patients, HHT did not affect relationship decisions (n = 353, 62%), decisions concerning pregnancy and children (n = 287, 50%) and sexual activity (n = 315, 57%). However, 28% of HHT patients (n = 157) did experience effect on sexual activity and may benefit from improved epistaxis management and better awareness of their partners.

**Conclusions:**

HHT did not affect family planning decisions and sexual activity in most patients, but approximately a quarter of patients experienced effect on sexual activity caused by epistaxis.

**Supplementary Information:**

The online version contains supplementary material available at 10.1186/s13023-025-03887-x.

## Background

Hereditary haemorrhagic telangiectasia (HHT) is a rare, dominantly inherited, vascular disease characterised by recurrent epistaxis, mucocutaneous telangiectases and visceral vascular malformations [1, 2]. It is a chronic disease with high variability in disease expression and severity [3]. Frequent symptoms include epistaxis, which can be severe and lead to iron deficiency anemia with subsequently fatigue and reduced exercise tolerance [1, 4].

Due to the genetic origin of the disease and the risk of (rare) major complications of pregnancy, the HHT diagnosis may influence family planning decisions [5]. Furthermore, previous research has presented the influence of HHT on the quality of life (QoL), which is mostly caused by epistaxis [6–11]. Also, daily lives and social relationships can be affected. The effect of HHT (including epistaxis) on family planning decisions, sexual activity and contraception has not been investigated to date.

The idea for this inventory survey study was born in the physician committee of the European Reference Network on rare multisystemic vascular diseases (VASCERN) and directly endorsed by the European Patient Advocacy Group (ePAG) and patient organisations. The objective of this study was to gain more insight into the patient’s needs on the subject of family planning, sexual activity and contraception in HHT patients.

## Methods

### Aim, design and setting

This was a prospective questionnaire-based investigation. The conception of the study was proposed by physicians of the VASCERN group, and endorsed by ePAG, with the aim of perceiving patient’s needs on family planning and intimacy topics. The questionnaire was carefully designed by Healthcare Providers (HCPs) of the HHT working group of VASCERN and the ePAG during an extensive developmental process and was thereafter approved by all members. The survey study was further developed at the St. Antonius Hospital, the Dutch HHT expertise center in Nieuwegein, the Netherlands.

The questionnaire was translated into ten languages: Danish, Dutch, English, Finnish, French, German, Italian, Norwegian, Spanish and Swedish. The translation was performed by native-speaking HHT experts (HCP and/ or member of the HHT-patient association) and included a forward translation (from English into the target language), a backward translation (the translated document back to English), a comparison of the translated document with the original English questionnaire and a discussion regarding the best suitable translation.

### Participant characteristics

HHT patients and their partners were informed about the study and invited to participate through the HHT expert centers during consultations, social media, HHT-newsletters and websites of European patient associations. Participating in the study was possible between March and May 2023. Information about the study was provided through a link and electronic informed consent was obtained from all participating patients and partners before entering the questionnaire.

### Ethical considerations

The questionnaires were fully anonymous, the researchers could not by any means identify patients who participated in the study. In cases where patients were directly invited by the center it was not possible to identify who accepted the invitation from those who did not. Based on the design and content of the study (including no possibility of patient identification), the Dutch law on Research Involving Human Subjects Act (WMO) was not applicable, as verified by the Medical Research Ethics Committees United (MEC-U; W22.132) and approved by the Local Research & Development department (Z22.085).

### Data collection

The questions included in the survey were divided into four different categories: general questions on HHT, family planning, sexual activity and contraception. Genetic confirmation of the diagnosis and the Curaçao criteria were included in the general questions of the survey to assess the presence of a definite clinical diagnosis.^13^ Three or more clinical criteria were required for a definite clinical diagnosis. The impression of the severity of epistaxis and HHT using a Visual Analogue Score (VAS) was included, range 0–10, with a higher number corresponding with more severe complaints. The contraception-specific questions were only visible to female patient participants. The complete survey was provided in both the patient’s version [see Additional file 1] and partner’s version [see Additional file 2]. The survey was designed in the data management system REDCap (Research Electronic Data Capture) hosted at the St. Antonius Hospital and could be accessed through a digital link. REDCap is a secure, web-based software platform designed to support data capture for research studies [12, 13]. Responses to the questionnaire were anonymous and not traceable back to the patient. No additional data were collected.

### Statistical analysis

Categorical data were presented as numbers (percentages). Reported answers can be found in Additional file 3 and 4. Continuous data were presented as mean (± standard deviation) or as median (interquartile ranges) in case data are not normally distributed. Subgroup-analyses were performed with age-groups, nationality-groups and groups based on the presence of visceral malformations (the latter only for the influence on decisions regarding pregnancy/children). Results from nationalities with more than 50 participants were shown by Group (French, Italian, Danish, Dutch and German). Nationalities with less than 50 participants (Spanish, British, Finnish, Swedish, Belgian, Norwegian, other nationality or no nationality reported) were included in the subgroup of ‘Other nationalities’. Data were visualized in grouped column charts. Comparison testing included chi-square test for additional investigation of a possible association between categorical variables and Kruskal–wallis test for the investigation of a possible association between a continuous non-normally distributed variable. Additional statistic testing included Cramer’s V coefficient for further interpretation of the strength of an association found with chi-square: a value of < 0.25 was considered as a weak association, 0.25–0.75 as a moderate association and > 0.75 as a strong association. Lastly, logistic regression was used to investigate the correlation between the VAS score of the HHT- and epistaxis severity and VAS score for the influence of HHT on sexual activity. For the exploration between subgroups (age/ nationality) and the VAS score for the influence of HHT on sexual activity, logistic regression in the form of a generalized linear model was used and included correction for the VAS-scores for HHT- and epistaxis severity. A p-value of < 0.05 was considered as statistically significant. The outcomes of the survey were analyzed using SPSS statistics version 29 for Windows (IBM, Armonk, NY, USA). Only relevant details were described in the manuscript, all statistical results from the subgroup-analyses are provided [see Additional file 5].

## Results

### General questions patients

**Fig. 1:**
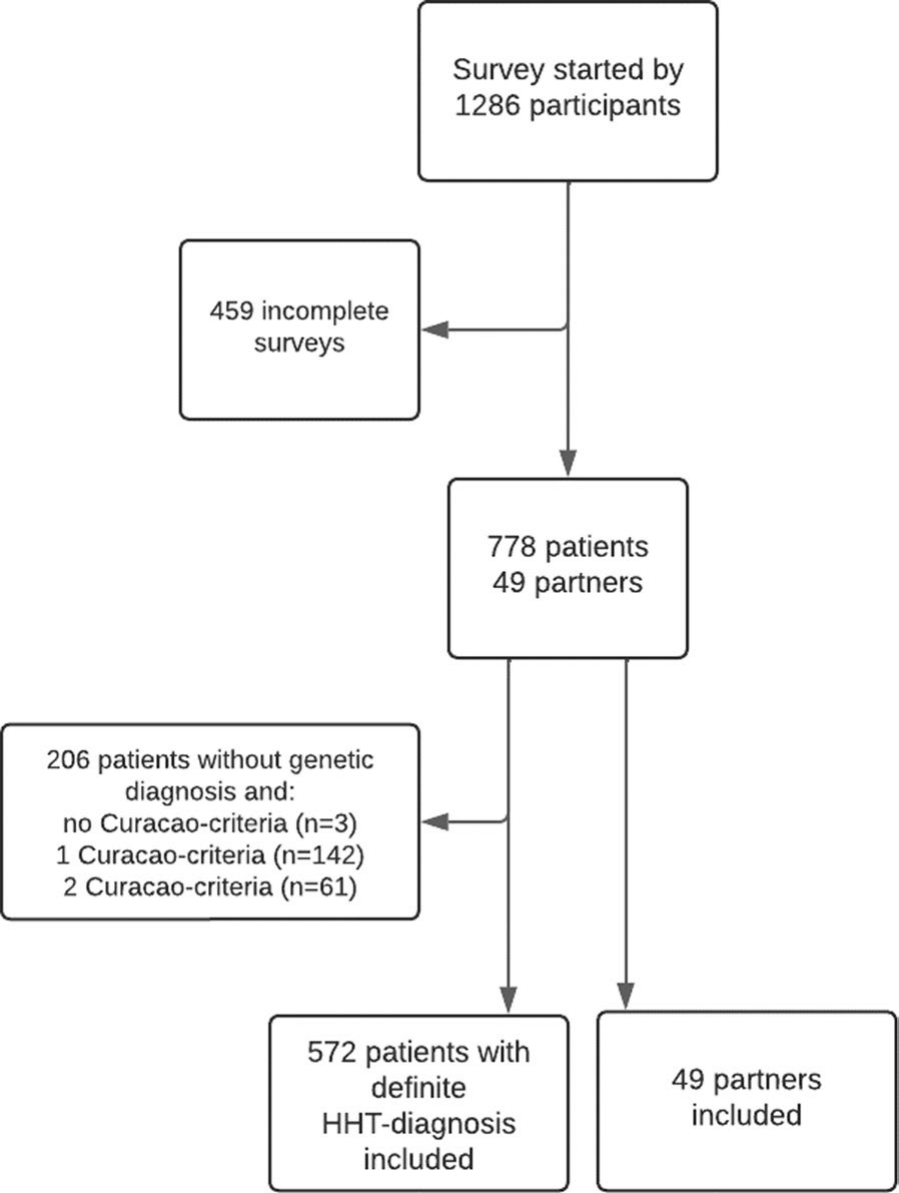
Inclusion flowchart

**Fig. 2:**
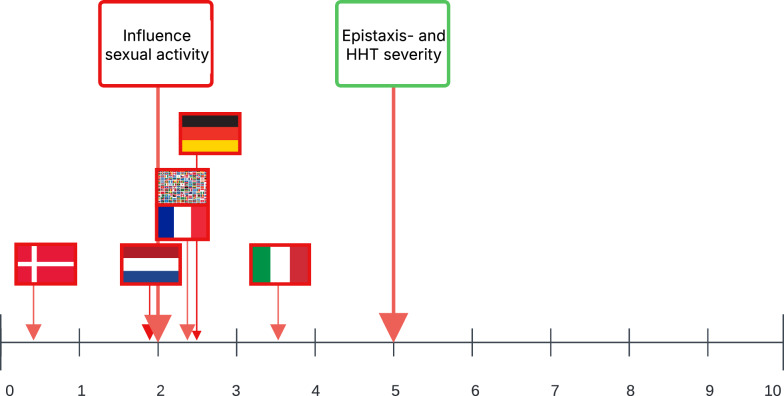
VAS-score (Visual Analogue Scale) influence of hereditary hemorrhagic telangiectasia complaints on sexual activity

The survey was completed by 778 patients, of which 572 participants (71% females, n = 406) met a definite HHT diagnosis and were included in the further analysis. A flowchart visualizing the inclusion details can be found in Fig. 1. Baseline characteristics can be found in Table 1. The majority of patients visited an HHT expert center at least once (n = 426; 75%). The patients reported a median VAS-score, scale 0 (not severe at all) to 10 (the most severe imaginable,) for the nosebleed severity of 5 (IQR 3.0–7.0) and a mean VAS-score, scale 0 (not severe at all) to 10 (the most severe imaginable), for the HHT severity of 5 (IQR 3.0–7.0). The reported VAS-score for the HHT and epistaxis severity was significantly different among the nationalities (p = 0.04 and p = 0.03), with the lowest median of 5 and 4 in Danish HHT patients (Fig. 2).

**Table 1:** Baseline characteristics patients

Included patients, n	572
Age, n(%)	
Under 25 years	17 (3)
25–35 years	73 (13)
35–45 years	114 (20)
45–55 years	102 (18)
55–65 years	142 (25)
65 years and older	124 (22)
Sex, n (%)	
Female	406 (71)
Male	164 (29)
Other	1 (0.2)
HHT type, n (%)	
HHT type 1	153 (27)
HHT type 2	186 (33)
SMAD 4	10 (2)
Unknown	182 (32)
VAS HHT severity, median (IQR)	5 (3.0–7.0)
VAS epistaxis severity, median (IQR)	5 (3.0–7.0)

### Family planning answers of patients

In most HHT patients (n = 353, 62%), there was no effect of HHT on the decisions concerning relationships. In 170 patients, there was only some minor concern and worry (30%). The effect of HHT on the decisions concerning pregnancy and children was not applicable in 287 patients (50%). HHT influenced the decision to have children in 91 patients (16%), to have fewer children in 43 patients (8%), not to have children in 40 patients (7%), to perform prenatal genetic testing for HHT in 32 patients (6%) and to perform embryonic selection to exclude HHT in 23 patients (4%). The most important contributing factors reducing the influence of HHT on family planning decisions were considered patient-friendly information in 153 patients (27%) and improved treatments for HHT in 148 patients (26%).

**Fig. 3:**
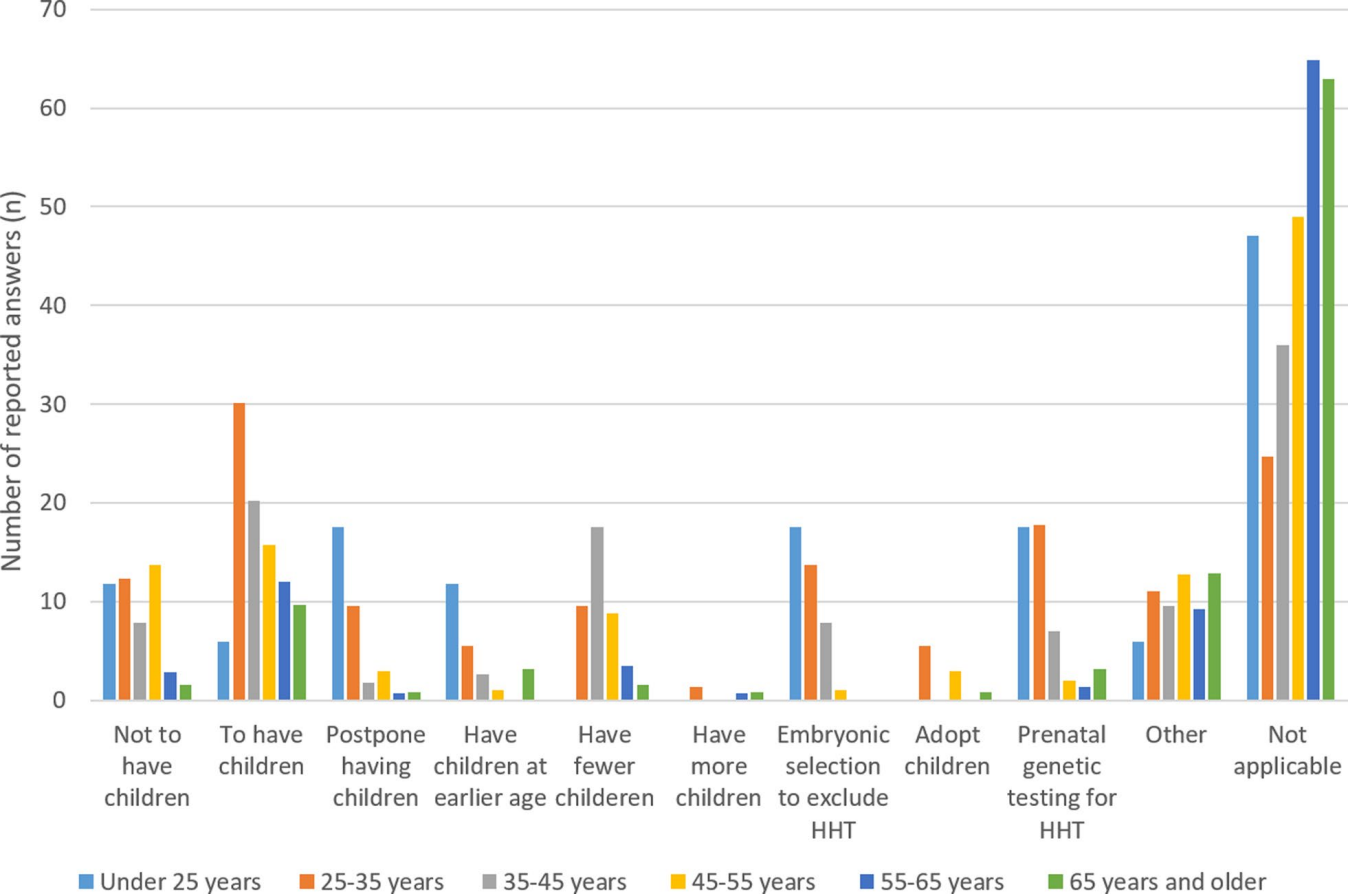
The influence of HHT on decisions regarding family planning: influence pregnancy and children among different age-groups

A moderate association was found between the age groups and the decision to perform embryonic selection to exclude HHT (*p* < 0.001 / Cramer’s V 0.28), which was more frequent reported in younger age groups, as visible in Fig. 3. Only (very) weak associations were found with the presence of (specific) visceral malformations.

The reported answers regarding family planning can be found in Table 2 and Additional file 3.

**Table 2:** Reported answers family planning patients

Influence HHT relationships*, n(%)	
Only some minor concern/ worry	170 (30)
Decided not to have a relationship	13 (2)
Decided to have a relationship	25 (4)
Decided to postpone relationship	3 (1)
There was no effect	353 (62)
Influence HHT pregnancy and children*, n(%)	
Not to have children	40 (7)
To have children	91 (16)
Postpone having children	17 (3)
Have children at earlier age	14 (2)
Have fewer children	43 (8)
Have more children	3 (1)
Embryonic selection to exclude HHT	23 (4)
Adopt children	8 (1)
Perform prenatal genetic testing for HHT	32 (6)
Other	62 (11)
Not applicable	287 (50)
Reduction influence HHT on family planning*, n(%)	
Patient-friendly information	153 (27)
Answers to my questions	105 (18)
Improved access to HHT-expert center	120 (21)
Improved treatments for HHT	148 (26)
Support for other HHT patients in my family	31 (5)
Support from other HHT patients in my family	26 (5)
Patient support groups	67 (12)
Economic support	39 (7)
Other	189 (33)
I don’t think anything would have helped	62 (11)

^*^multiple answers could be reported by a single patient

### Sexual activity answers of patients

57% of HHT patients (n = 315) did not think having HHT influenced their current, potential or previous intimacy and sexual activity. 157 patients (28%) reported an influence and 84 patients (15%) did not know. The VAS score (scale 0–10) of the influence of HHT complaints on sexual activity was a median of 2 (IQR 0–6). The most frequent experienced emotion was fear of having HHT symptoms in 171 patients (30%). The most common associated symptom was epistaxis (in 252 patients, 44%). The most important contributing factor for a reduction of the influence was considered partners who make the patient feeling comfortable (n = 217, 38%).

**Fig. 4:**
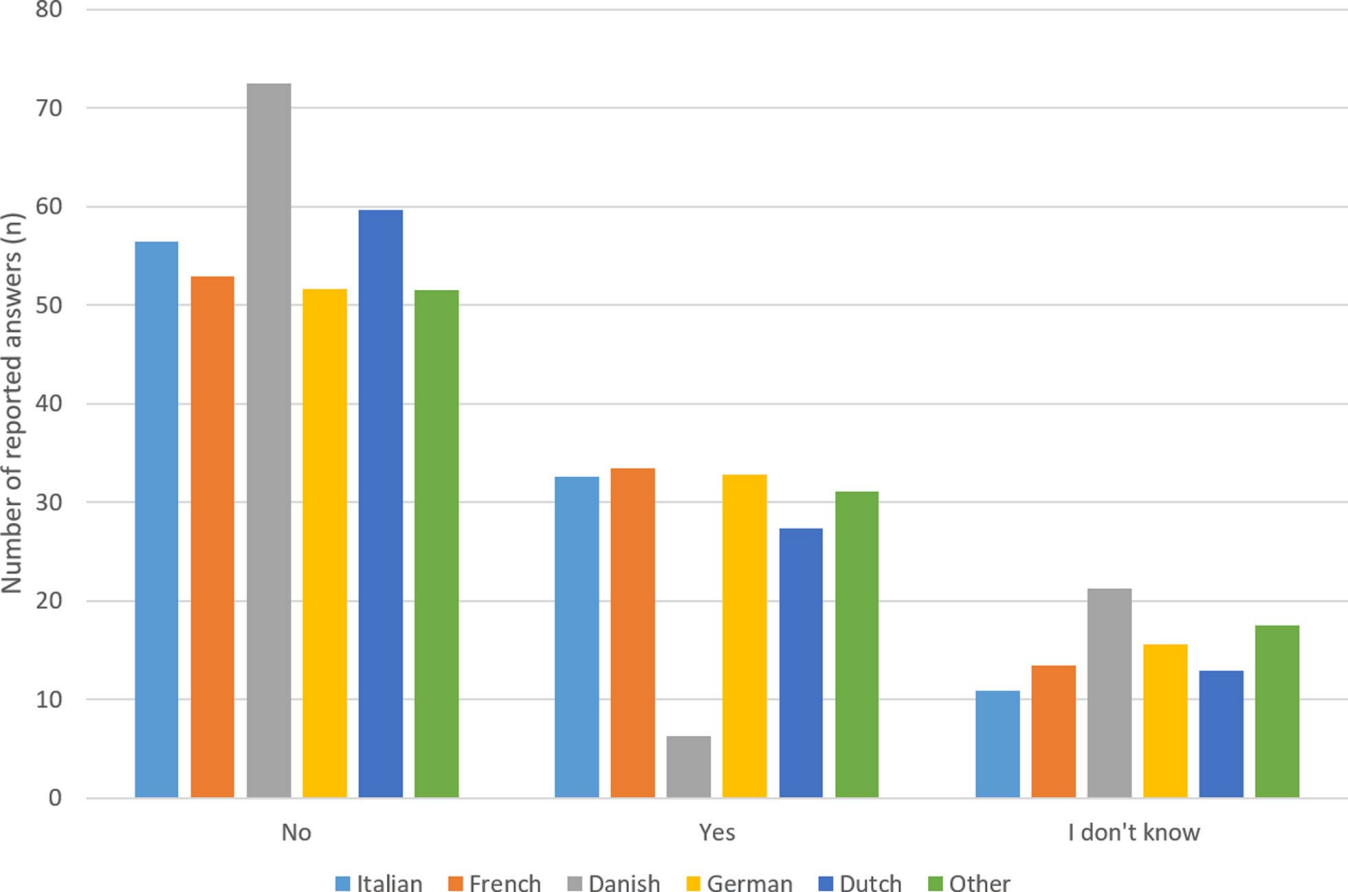
The influence of HHT on sexual activity among different nationalities

A significant correlation was found between the VAS for HHT- and epistaxis severity and the VAS influence of HHT complaints on sexual activity with a *p*-value < 0.001 and 0.003 and OR of 1.45 (95% CI 1.28–1.64) and 1.19 (95% CI 1.06–1.34). Furthermore, participants with a Danish nationality experienced less influence of HHT on sexual activity (as visible in Fig. 4); a correlation was also found with the Danish nationality with a p-value of 0.009 (corrected for age, HHT- and epistaxis severity) and an OR of 0.34 (95% CI 0.15–0.77). The median VAS for the HHT- and epistaxis severity (in green), the median VAS influence of HHT complaints on sexual activity (in red) and VAS per nationality (red) were visualized in Fig. 2 with a range of 0 (no influence at all) to 10 (the biggest influence imaginable).

The reported answers regarding sexual activity can be found in Table 3 and in Additional file 3.

**Table 3:** Reported answers sexual activity patients

Emotions in sexual life because of HHT symptoms*, n(%)	
Distress	70 (12)
Frustration	85 (15)
Sexual inadequacy	30 (5)
Dissatisfaction	39 (7)
Bothered by low sexual desire	77 (14)
Embarrassment	91 (16)
Fear of having HHT symptoms	171 (30)
None of the above	272 (48)
Which symptoms*, n(%)	
Epistaxis	252 (44)
Bleeding elsewhere	63 (11)
Fatigue	118 (21)
Shortness of breath	96 (17)
Reduced exercise tolerance	70 (12)
Palpitations	56 (10)
Epilepsy	9 (2)
Headache/ migraine	64 (11)
Consequence of these emotions*, n(%)	
Low sexual desire	97 (17)
Avoid sexual activity occasionally	84 (15)
Avoid sexual activity in general	25 (4)
Avoid having relationships	29 (5)
Reduction of influence*, n(%)	
Not symptomatic	53 (9)
Only mild symptoms	171 (30)
No (more) symptoms during intimacy/ sexual activity	116 (20)
Very comfortable with HHT	72 (13)
Partner(s) aware of HHT and make comfortable	217 (38)
More information necessary, n(%)	232 (41)

^*^multiple answers could be reported by a single patient

### Contraception answers of patients

Hormonal contraception was the most used method of contraception in female patients with HHT, used by 259 patients (64%), followed by barrier methods (n = 171, 42%), hormonal intra-uterine device (IUD; n = 77, 19%), copper IUD (n = 57, 14%), natural contraceptives (n = 40, 10%), sterilization (n = 23, 6%) and other (n = 19, 5%). 29 patients had never used contraception (7%).

The majority of patients with HHT reported they were not informed at all about the options and benefits, and disadvantages of the specific types of contraceptives (n = 219, 54%).

### Partners outcomes

49 partners of HHT patients completed the questionnaire, including 29 females (59%) and the majority with an age between 45 and 65 years (n = 27, 55%). The participants consisted of 17 partners of patients with an ENG mutation (HHT type 1) (35%), 16 partners of patients with an ACVRL1 mutation (HHT type 2) (35%), 1 partner of a SMAD4 patient (2%) and 13 with an unknown type of HHT (28%). Partners had a perception of the patient’s nosebleeds with a median VAS score (scale 0–10) of 5.0 (IQR 3.0–7.0) and median VAS score (scale 0–10) for severity of HHT of 6(IQR 5.0–7.0).

In the 35 (71%) of the partners of HHT patients there was no effect of HHT on relationship decisions. In 11 partners, there was only some minor concern and worry (22%). In 55% of the partners (n = 27), the influence of HHT on the pregnancy/ children decision was not applicable. Improved treatments for HHT were considered the most important contributor to reducing the influence on family planning, reported by 19 partners (39%), followed by patient-friendly information by 15 partners (31%) and answers to my questions by 14 partners (29%).

29 partners (59%) reported no influence of HHT on intimacy and sexual activity. The median VAS score (scale 0–10) for the influence of HHT on sexual activity was 2 (IQR 0.2–6.1). Most contributable for a reduction of the influence, was considered the awareness of the partner who (tried to make) made the HHT-patient feel comfortable about their HHT (51%, reported by 25 partners), followed by no more symptoms occurring during intimate activities (n = 15, 31%). These reported answers can be found in Additional file 4.

## Discussion

This European survey study investigated the subject of family planning, sexual activity and contraception in HHT patients with a very high number of respondents. Results of this study highlights the importance of discussing this issue with patients during consultations. In most patients with HHT there was no effect of HHT on family planning decisions and sexual activities. A minority of the patients deemed that this effect on family planning decisions could have been reduced by providing patient-friendly information, improved treatments for HHT and support for HHT patients in their family.

Previous studies have demonstrated that recurrent epistaxis is the leading cause of morbidity in HHT patients, associated with emotional consequences, decreased physical and social functioning, and fatigue complaints.[2, 6, 9–11]. The impact of HHT on the QoL of patients was studied for the first time in 2004 by Pasculli et al. using the Short Form-36 Health Survey [10]. Worse scores were found in HHT-patients, determined by physical health, mental and general health. Specifically epistaxis was found to have a negative impact on the QoL. This finding of recurrent epistaxis as the leading cause for a decrease in QoL was confirmed in later studies [6–8, 14–17]. It was associated with emotional consequences, decreased physical functioning, fatigue and social functioning. Besides the use of questionnaires to gain insight, Martinent et al. performed semi-structured interviews in patients with HHT to get a more accurate description of health-related QoL. The QoL was considered to be closely determined by the health state, with the greatest impact of epistaxis and fatigue complaints (consistent with the literature) [9]. More recently, Le et al. developed an HHT-specific QoL-questionnaire, the QoL-HHT (24 items), including six HHT-specific domains: physical limitations, social relationships, concern about bleeding, relationship with the medical profession, experience of symptoms and concern about the evolution of the disease [18]. A shorter HHT-specific QoL instrument has been developed by Kasthuri et al. specifically assessing whether HHT-symptoms interfered with social interactions [19].

We found that there is little to no influence of HHT on relationship decisions reported, which could be influenced by the age of the participants (> 45 years in 65%) and the time of diagnosis. For the influence on pregnancy and getting children, we see no clear trend between the ‘positive’ (to have children, more, earlier) and ‘negative’ (not to have, less, postpone) answers. Complicating the analysis was the possibility to report multiple answers by a single participant. As expected, it was found that the decision to perform embryonic selection was more frequent reported in younger age groups—which could be explained by both earlier HHT-diagnosis (due to increased awareness, increased screening of family members) and emerging developments in preimplantation genetic testing (PGT). Although more used in general[20], the option is not widely used in HHT yet and was only considered valuable in a few cases [2].

In a small questionnaire-based study from Geisthoff et al., 16/60 patients (27%) reported effect of HHT on family planning—which is consistent with our findings [6].

In line with the results of previous studies on the influence on the QoL, we found that epistaxis complaints are most frequently reported to influence sexual activity in up to a quarter of the HHT patients. Interestingly, it was the awareness of partners which was considered most beneficial for a reduction of the influence (instead of the severity of the symptoms or the increase during sexual activity). On the basis of the survey outcomes, we could not distinguish if either the fear of having epistaxis influences sexual activity or the actual occurring of epistaxis. This is important to specify in the outpatient clinic, to determine on which domain improvement can be achieved (either awareness and/ or epistaxis management). With the results of this study, further meetings will be scheduled with the ePAG and patient associations for the development of a strategy. Additional research with validated questionnaires is necessary to confirm the results.

The strength of this study is that to our knowledge no studies have been published on the subject on the subject of family planning, sexual activity and contraception in HHT. Secondly, there is a (very) large number of respondents with a rare disease, with a re-confirmed HHT diagnosis based on the inclusion of the Curaçao-criteria in the general section of the survey. Further strengths of the study include the complete design of the questionnaire in collaboration with both HHT experts and HHT patients and the high-quality translations in ten different languages resulting in accessibility of the survey.

There are several limitations of this study. Most importantly, the study was designed as an inventory study, with a custom-made questionnaire. Second, there is an important risk of selection bias due to the inclusion-method. Patients were not randomly selected, but invitations for the survey were allowed through social media and patients associations. Also, the participating patients were mostly from countries with an HHT reference center part of the VASCERN: France, Italy, Denmark, Germany and the Netherlands—which might influence the external validity of the results. Third, there is a risk of recall-bias. For example, the survey respondents includes 47% of patients aged 55 years and older, for whom it is most likely that decisions concerning pregnancy/ children were made years ago and often even before they were diagnosed with an informed about HHT. Furthermore, the Danish participants reported a significant lower severity of the disease, which might have influenced the subgroup analysis (except for the VAS-score on the influence on sexual activity, where it was possible to correct for the difference in HHT severity scores). Additionally, the method of recruitment differed between the centers and differences in cultures and countries could play a role in the experience of the effects of the disease, for example the circumstance that not in all countries embryonic selection is legal. Another limitation, is not including the age/ time of diagnosis in the survey and not including male patients in the contraception-part of the survey. We did not expect impact of the disease for condom / sterilization use in male patients, therefore consensus was to not include this section for the male-version of the survey, but we cannot rule out any impact. Lastly, not applicable in the section of influence on family planning may include different interpretations of that answer: not applicable since not involved in family planning decisions, or the influence of HHT on family planning decisions is not applicable, or the HHT-diagnosis was not known at the time of family planning decisions. The results would need further validation.

## Conclusions

The results of our study show that in most patients, HHT does not affect family planning decisions and sexual activity. However, a non-negligible number of HHT patients (28%) did experience effect on sexual activity and may benefit from improved epistaxis management, better awareness of their partners and adequate counselling by HHT experts.

## Supplementary Information

Additional file 1: Partner survey. This file includes the introductory text, the general questions, questions concerning family planning, sexual activity and contraception.

Additional file 2: Partner survey. This file includes the introductory text, the general questions, questions concerning family planning and sexual activity.

Additional file 3: Reported answers patient participants. This file includes the numbers of reported answers (raw data) on the complete survey by patient participants.

Additional file 4: Reported answers partner participants. This file includes the numbers of reported answers (raw data) on the complete survey by partner participants.

Additional file 5: Subgroup analysis statistical analysis. This file includes the statistical results from the subgroup analysis (based on age and nationality) including chi-square test results, and in case of a significant association also Cramer’s V coefficient for further interpretation.

## Data Availability

The data supporting the conclusions of this article are included within the article and its additional files.
