# Supplementary material for: Family planning, sexual activity and contraception in hereditary hemorrhagic telangiectasia: a European survey study
Source: Orphanet J Rare Dis. 2025 Aug 1;20:395. doi: 10.1186/s13023-025-03887-x (PMC12317622; doi:10.1186/s13023-025-03887-x)
Supplement: Supplementary file 1 — Additional file 1: Partner survey. This file includes the introductory text, the general questions, questions concerning family planning, sexual activity and contraception. [file 13023_2025_3887_MOESM1_ESM.pdf]

Additional file 1. Patient survey

Family planning, intimacy, sexual activity and contraception in hereditary haemorrhagic telangiectasia

Hello! This is an anonymous survey for patients with hereditary hemorrhagic telangiectasia (HHT) and their partners concerning the subjects of family planning, intimacy and sexual activity and contraception. This survey was commissioned by the VASCERN, the European HHT Reference Network. The aim of this survey is that we would like to know more about the patients' needs to further improve the care for HHT patients.

Please complete this 15-minute survey. Your responses are anonymous and you can skip any question you are not comfortable with answering. Your responses will be stored in Redcap and will not be shared with commercial companies. Data analysis will take place in the St. Antonius Hospital in Nieuwegein, the Netherlands. Data could be used for scientific publication, in which the responses are not traceable back to you. The same link can be used by different patients and their partners. Thank you for your participation.

Do you agree with the use of your answers in the survey for scientific purposes?

- Yes
- No

General questions

What is your age?
- Under 25 years
- 25-35 years
- 35-45 years
- 45-55 years
- 55-65 years
- 65 years or older

What is your Nationality?
- Italian
- French
- Danish
- German
- Dutch
- Spanish
- British
- Finnish
- Swedish
- Belgian
- Norwegian
- Other

Which country do you currently live in?
- Austria
- Belgium
- Bulgaria
- Croatia
- Republic of Cyprus
- Czech Republic
- Denmark

- Estonia
- Finland
- France
- Germany
- Greece
- Hungary
- Ireland
- Italy
- Latvia
- Lithuania
- Luxembourg
- Malta
- Netherlands
- Norway
- Poland
- Portugal
- Romania
- Slovakia
- Slovenia
- Spain
- Sweden
- Other

What is your sex?

- Female
- Male
- Diverse
- I'd prefer not to say

Do you have or your partner has hereditary haemorrhagic telangiectasia (HHT)?

- I have HHT
- My partner has HHT (see appendix B. Partners survey)

How was your HHT diagnosed? Please check all boxes applicable to you.

- ○ Spontaneous recurrent nosebleeds
- ○ Multiple telangiectases/ red spots on the skin of the hands, lips, face, inside mouth or nose
- ○ Abnormal blood vessels in one of the organs (brain, lungs, liver, intestines)
- ○ First-degree relative with HHT
- ○ Genetically confirmed diagnosis

Do you know what gene/ type causes your HHT?

- Endoglin (HHT type 1)
- ACVRL1 (HHT type 2)
- SMAD4
- I don't know/ no mutation found/ no genetic testing performed/ ongoing analysis/ other

Do you have vascular abnormalities in any of your organs? (treated as well as untreated)

- Yes
- No

- I don't know

Yes, in the:
  - Lungs
  - Liver
  - Stomach and/ or intestines
  - Brain
  - Other

In the: …. (open text box)

Have you visited the hospital in the last 6 months because of HHT-related bleeding?
- Yes
- No

What is your perception on your nosebleeds?
not severe at all ▬▬▬▬▬▬▬▬▬▬ the most severe imaginable

Change the slider above to set a response

What is your perception on the severity of your HHT?
not severe at all ▬▬▬▬▬▬▬▬▬▬ the most severe imaginable

Change the slider above to set a response

Have you ever been to an HHT expert center?
- Yes
- Yes, only for genetic counselling
- No

How did you receive this questionnaire?
- Patient association
- HHT expert center
- Newsletter
- Social media
- Family member
- Other

Family planning

In what way did HHT affect your decisions concerning relationships?
  - Only some minor concern and worry
  - Decided not to have a relationship
  - Decided to have a relationship
  - Decided to postpone relationship until after diagnosis
  - Decided to postpone relationship until after screening and/or treatment
  - There was no effect

In what way did HHT affect your decisions concerning pregnancy and children?
HHT probably influenced my/ our decision to:
  - Not to have children
  - Have children

- Postpone having children
- Have children at earlier age
- Have fewer children
- Have more children
- Embryonic selection to exclude HHT
- Adopt children
- Perform prenatal genetic testing for HHT
- Other
- Not applicable

What do you think helped/ would have helped you reduce the influence of your (partner's) HHT on your family planning?

- Patient-friendly information
- Answers to my questions
- Improved access to an HHT expert center
- Improved treatments for HHT
- Support for other HHT patients in my family that I care for
- Support from other HHT patients in my family
- Patient support groups
- Economic support
- I don't think anything would have helped me/ us
- Other

Intimacy and sexual activity

Do you consider yourself sexually active?

- Yes
- No

Do you think having HHT has influenced your current/ potential/ previous intimacy and sexual activity?

- Yes
- No
- I don't know

Have you ever experienced the following emotions in your sexual life because of HHT symptoms?

- Distress
- Frustration
- Sexual inadequacy
- Dissatisfaction
- Bothered by low sexual desire
- Embarrassment
- Fear of having HHT symptoms
- Other
- None of the above

Which symptoms?

- Nosebleeds
- Bleeding from somewhere else
- Fatigue
- Shortness of breath
- Reduced exercise tolerance
- Palpitations
- Epilepsy
- Headache/ migraine

- Other

What is the consequence of these emotions?

- Low sexual desire
- Avoid sexual activity occasionally
- Avoid sexual activity in general
- Avoid having relationships
- Other

How would you classify the influence of your HHT complaints on your sexual activity?
no influence at all the biggest influence imaginable
Change the slider above to set a response

What contributes to a reduction of the influence of your HHT on your intimacy and sexual activity?

- I am not symptomatic
- I only have mild symptoms
- I don't have (more) symptoms during intimacy or sexual activities
- I feel very comfortable with my HHT
- My partner(s) have been aware of my HHT and make me feel comfortable about it
- Other

Do you think it is necessary/ important that there is more attention regarding the influence of HHT on intimacy and sexual activity?

- Yes, I think that is necessary.
- No, it's not necessary
- I don't know

Contraception

Which type(s) of contraceptives did you ever use?

- I have never used contraception
- Condoms, female condom, diaphram/cap
- Hormonal contraception (pill, rod, ring, injection)
- Hormonal intra-uterine device (IUD)
- Copper IUD
- Natural contraceptive/ natural family planning (preventing pregnancy without the use of medications or devices; for example calendar rhythm method, basal body temperature method, cervical mucus examination, herbs)
- Sterilization
- Other

Please list the name of the pill: …. (open text box)

Do you think you were well informed about the options and their respective pros and cons before starting with contraceptives?

- Yes
- No, I was not informed at all
- I was informed, but I missed something

Who supplied you with the information?

- The general practitioner/ family doctor
- Gynaecologist
- HHT-specialized medical specialist

- Other medical specialist
- Internet
- Family/ friends
- Other

Family planning, sexual activity and contraception in hereditary hemorrhagic telangiectasia: a European survey study, Orphanet Journal of Rare Diseases, J. Hessels et al., pulmonary department St. Antonius Hospital, j.hessels@antoniusziekenhuis.nl
