# Supplementary material for: Family planning, sexual activity and contraception in hereditary hemorrhagic telangiectasia: a European survey study
Source: Orphanet J Rare Dis. 2025 Aug 1;20:395. doi: 10.1186/s13023-025-03887-x (PMC12317622; doi:10.1186/s13023-025-03887-x)
Supplement: Supplementary file 2 — Additional file 2: Partner survey. This file includes the introductory text, the general questions, questions concerning family planning and sexual activity. [file 13023_2025_3887_MOESM2_ESM.pdf]

Additional file 2. Partner survey

Family planning, intimacy, sexual activity and contraception in hereditary haemorrhagic telangiectasia

Hello! This is an anonymous survey for patients with hereditary hemorrhagic telangiectasia (HHT) and their partners concerning the subjects of family planning, intimacy and sexual activity and contraception. This survey was commissioned by the VASCERN, the European HHT Reference Network. The aim of this survey is that we would like to know more about the patients' needs to further improve the care for HHT patients.

Please complete this 15-minute survey. Your responses are anonymous and you can skip any question you are not comfortable with answering. Your responses will be stored in Redcap and will not be shared with commercial companies. Data analysis will take place in the St. Antonius Hospital in Nieuwegein, the Netherlands. Data could be used for scientific publication, in which the responses are not traceable back to you. The same link can be used by different patients and their partners. Thank you for your participation.

Do you agree with the use of your answers in the survey for scientific purposes?

- Yes
- No

General questions

What is your age?

- Under 25 years
- 25-35 years
- 35-45 years
- 45-55 years
- 55-65 years
- 65 years or older

What is your Nationality?

- Italian
- French
- Danish
- German
- Dutch
- Spanish
- British
- Finnish
- Swedish
- Belgian
- Norwegian
- Other

Which country do you currently live in?

- Austria
- Belgium
- Bulgaria
- Croatia
- Republic of Cyprus
- Czech Republic
- Denmark

- Estonia
- Finland
- France
- Germany
- Greece
- Hungary
- Ireland
- Italy
- Latvia
- Lithuania
- Luxembourg
- Malta
- Netherlands
- Norway
- Poland
- Portugal
- Romania
- Slovakia
- Slovenia
- Spain
- Sweden
- Other

What is your sex?
- Female
- Male
- Diverse
- I'd prefer not to say

Do you have or your partner has hereditary haemorrhagic telangiectasia (HHT)?
- I have HHT (see appendix A. Patient survey)
- My partner has HHT

Do you know what gene/ type causes your partner's HHT?
- Endoglin (HHT type 1)
- ACVRL1 (HHT type 2)
- SMAD4
- I don't know/ no mutation found/ no genetic testing performed/ ongoing analysis/ other

Does your partner have vascular abnormalities in any of your organs? (treated as well as untreated)
- Yes
- No
- I don't know

Yes, in the:
- o Lungs
- o Liver
- o Stomach and/ or intestines
- o Brain
- o Other

In the: …. (open text box)

Did your partner visit the hospital in the last 6 months because of HHT-related bleeding?

- Yes
- No

What is your perception on your partner's nosebleeds?
not severe at all ▬▬▬▬▬▬▬▬▬▬ the most severe imaginable

Change the slider above to set a response

What is your perception on the severity of your partner's HHT?
not severe at all ▬▬▬▬▬▬▬▬▬▬ the most severe imaginable

Change the slider above to set a response

Have your partner ever been to an HHT expert center?

- Yes
- Yes, only for genetic counselling
- No
- I don't know

How did you receive this questionnaire?

- Patient association
- HHT expert center
- Newsletter
- Social media
- Family member
- Other

Family planning

In what way did HHT affect your decisions concerning relationships?

- Only some minor concern and worry
- Decided not to have a relationship
- Decided to have a relationship
- Decided to postpone relationship until after diagnosis
- Decided to postpone relationship until after screening and/or treatment
- There was no effect

In what way did HHT affect your decisions concerning pregnancy and children?
HHT probably influenced my/ our decision to:

- Not to have children
- Have children
- Postpone having children
- Have children at earlier age
- Have fewer children
- Have more children
- Embryonic selection to exclude HHT
- Adopt children
- Perform prenatal genetic testing for HHT
- Other
- Not applicable

What do you think helped/ would have helped you reduce the influence of your (partner's) HHT on your family planning?

- Patient-friendly information
- Answers to my questions
- Improved access to an HHT expert center
- Improved treatments for HHT
- Support for other HHT patients in my family that I care for
- Support from other HHT patients in my family
- Patient support groups
- Economic support
- I don't think anything would have helped me/ us
- Other

Intimacy and sexual activity

Do you consider yourself sexually active?

- Yes
- No

Do you think your partner’s HHT has influenced your current/ potential/ previous intimacy and sexual activity?

- Yes
- No
- I don't know

Have you ever experienced the following emotions in your sexual life because of your partner’s HHT symptoms?

- Distress
- Frustration
- Sexual inadequacy
- Dissatisfaction
- Bothered by low sexual desire
- Embarrassment
- Fear of your partner having HHT symptoms
- Other
- None of the above

Which symptoms?

- Nosebleeds
- Bleeding from somewhere else
- Fatigue
- Shortness of breath
- Reduced exercise tolerance
- Palpitations
- Epilepsy
- Headache/ migraine
- Other

What is the consequence of these emotions?

- Low sexual desire
- Avoid sexual activity occasionally
- Avoid sexual activity in general
- Avoid having relationships
- Other

How would you classify the influence of your partner's HHT complaints on your sexual activity?
no influence at all the biggest influence imaginable
Change the slider above to set a response

What contributes to a reduction of the influence of your partner's HHT on your intimacy and sexual activity?

- My partner is not symptomatic
- My partner only has mild symptoms
- My partner doesn't have (more) symptoms during intimacy or sexual activities
- My partner feels very comfortable with his/her/their HHT
- I am aware of my partner's HHT and (try to) make my partner feel comfortable about it
- Other

Do you think it is necessary/ important that there is more attention regarding the influence of HHT on intimacy and sexual activity?

- Yes, I think that is necessary.
- No, it's not necessary
- I don't know

Family planning, sexual activity and contraception in hereditary hemorrhagic telangiectasia: a

European survey study, Orphanet Journal of Rare Diseases, J. Hessels et al., pulmonary department

St. Antonius Hospital, j.hessels@antoniusziekenhuis.nl
