# Supplementary material for: Family planning, sexual activity and contraception in hereditary hemorrhagic telangiectasia: a European survey study
Source: Orphanet J Rare Dis. 2025 Aug 1;20:395. doi: 10.1186/s13023-025-03887-x (PMC12317622; doi:10.1186/s13023-025-03887-x)
Supplement: Supplementary file 3 — Additional file 3: Reported answers patient participants. This file includes the numbers of reported answers (raw data) on the complete survey by patient participants. [file 13023_2025_3887_MOESM3_ESM.pdf]

Additional file 3. Overview of reported answers patient participants

	All nationalities*	French*	Italian	Danish	German	Dutch	Other*	p-value
Included participants, n	572	161 (28)	94 (16)	83 (15)	64 (11)	64 (11)	106 (19)	
Age, n (%)								0.067
Under 25 years	17 (3)	2 (1)	2 (2)	6 (7)	2 (3)	4 (6)	1 (1)	
25-35 years	73 (13)	18 (11)	9 (10)	12 (14)	8 (13)	9 (14)	17 (16)	
35-45 years	114 (20)	32 (20)	13 (14)	13 (16)	17 (27)	12 (19)	27 (25)	
45-55 years	102 (18)	24 (15)	25 (27)	15 (18)	10 (16)	8 (13)	20 (19)	
55-65 years	142 (25)	42 (26)	28 (30)	13 (16)	15 (23)	18 (28)	26 (25)	
65 years and older	124 (22)	43 (27)	17 (18)	24 (29)	12 (19)	13 (20)	15 (14)	
Sex, n (%)								0.108
Female	406 (71)	113 (70)	63 (67)	51 (61)	54 (84)	48 (75)	77 (73)	
Male	164 (29)	48 (30)	30 (32)	31 (37)	10 (16)	16 (25)	29 (27)	
Other	1 (0.2)	0	0	1 (1)	0	0	0	
Missing	1 (0.2)	0	1 (1)	0	0	0	0	
HHT type, n (%)								0.223
HHT type 1	153 (27)	30 (19)	27 (29)	21 (25)	18 (28)	19 (30)	38 (36)	
HHT type 2	186 (33)	56 (35)	33 (35)	28 (34)	15 (23)	25 (39)	29 (27)	
SMAD 4	10 (2)	5 (3)	1 (1)	1 (1)	2 (3)	0	1 (1)	
Unknown	182 (32)	55 (34)	21 (22)	28 (34)	23 (36)	18 (28)	37 (35)	
Missing	41 (7)	15 (9)	12 (13)	5 (6)	6 (9)	2 (3)	1 (1)	
VAS HHT severity, median (IQR)	5 (3.0-7.0)	5 (4.0-7.0)	6 (4.0-7.0)	5 (2.0-6.0)	6 (3.0-7.0)	5 (3.0-7.0)	5 (3.0-7.0)	0.043[∞]
Missing, n (%)	67	21	12	12	6	2	14	
VAS epistaxis severity, median (IQR)	5 (3.0-7.0)	5 (3.0-7.0)	5 (3.0-7.0)	4 (1.5-6.0)	5 (3.0-7.0)	5 (2.0-7.0)	5 (3.0-7.0)	0.026[∞]
Missing, n (%)	24	7	5	2	3	1	6	
Received questionnaire through:								<0.001[∞]
Patient association	230 (40)	86 (53)	50 (53)	15 (18)	14 (22)	9 (14)	56 (53)	
HHT expert center	177 (31)	45 (28)	12 (13)	59 (71)	5 (8)	33 (52)	23 (22)	
Newsletter	22 (4)	2 (1)	8 (9)	2 (2)	0	3 (5)	7 (7)	
Social media	79 (14)	10 (6)	13 (14)	3 (4)	29 (45)	8 (13)	16 (15)	
Family member	23 (4)	6 (4)	5 (5)	0	3 (5)	9 (14)	0	
Other	23 (4)	9 (6)	0	3 (4)	10 (16)	0	1 (1)	
Missing	18 (3)	3 (2)	6 (6)	1 (1)	3 (5)	2 (3)	3 (3)	

Table 1. Baseline characteristics patients

*3 patients with a double-nationality (French and other) were included in the French-group, 2 patients did not report their nationality and were included in the other-nationality group.
[∞] statistically significant with a p-value < 0.05

Participants, n	572
Influence HHT relationships*, n(%)	
Only some minor concern/ worry	170 (30)
Decided not to have a relationship	13 (2)
Decided to have a relationship	25 (4)
Decided to postpone relationship until diagnosis	0
Decided to postpone relationship until after screening/ treatment	3 (1)
There was no effect	353 (62)
Influence HHT pregnancy and children*, n(%)	
Not to have children	40 (7)
To have children	91 (16)
Postpone having children	17 (3)
Have children at earlier age	14 (2)
Have fewer children	43 (8)
Have more children	3 (1)
Embryonic selection to exclude HHT	23 (4)
Adopt children	8 (1)
Perform prenatal genetic testing for HHT	32 (6)
Other	62 (11)
Not applicable	287 (50)
Reduction influence HHT on family planning*, n(%)	
Patient-friendly information	153 (27)
Answers to my questions	105 (18)
Improved access to HHT-expert center	120 (21)
Improved treatments for HHT	148 (26)
Support for other HHT patients in my family	31 (5)
Support from other HHT patients in my family	26 (5)
Patient support groups	67 (12)
Economic support	39 (7)
Other	189 (33)
I don't think anything would have helped	62 (11)

Table 2. Reported answers family planning patients
* multiple answers could be reported by a single patient

Included participants (n)	572
Sexually active, n (%)	388 (68)
Missing	16 (3)
Influence HHT on sexual activity, n(%)	
Yes	157 (27)
No	315 (55)
I don't know	84 (15)
Missing	16 (3)
Emotions in sexual life because of HHT symptoms*, n(%)	
Distress	70 (12)
Frustration	85 (15)
Sexual inadequacy	30 (5)
Dissatisfaction	39 (7)
Bothered by low sexual desire	77 (14)
Embarrassment	91 (16)
Fear of having HHT symptoms	171 (30)
Other	7 (1)
None of the above	272 (48)

Which symptoms*, n(%)	
Epistaxis	252 (44)
Bleeding elsewhere	63 (11)
Fatigue	118 (21)
Shortness of breath	96 (17)
Reduced exercise tolerance	70 (12)
Palpitations	56 (10)
Epilepsy	9 (2)
Headache/ migraine	64 (11)
Other	9 (2)
Included participants (n)	572
Consequence of these emotions*, n(%)	
Low sexual desire	97 (17)
Avoid sexual activity occasionally	84 (15)
Avoid sexual activity in general	25 (4)
Avoid having relationships	29 (5)
Other	69 (12)
VAS influence HHT complaints on sexual activity, median (IQR)	2 (0.4-5.7)
Missing, n (%)	53 (9)
Reduction of influence*, n(%)	
Not symptomatic	53 (9)
Only mild symptoms	171 (30)
No (more) symptoms during intimacy/ sexual activity	116 (20)
Very comfortable with HHT	72 (13)
Partner(s) aware of HHT and make comfortable	217 (38)
Other	49 (9)
More information necessary, n(%)	
No, it's not necessary	104 (18)
Yes, it's necessary	232 (41)
I don't know	224 (39
Missing	12 (2)

Table 3. Reported answers sexual activity patients

Family planning, sexual activity and contraception in hereditary hemorrhagic telangiectasia: a European survey study, Orphanet Journal of Rare Diseases, J. Hessels et al., pulmonary department St. Antonius Hospital, j.hessels@antoniusziekenhuis.nl
