# Supplementary material for: Family planning, sexual activity and contraception in hereditary hemorrhagic telangiectasia: a European survey study
Source: Orphanet J Rare Dis. 2025 Aug 1;20:395. doi: 10.1186/s13023-025-03887-x (PMC12317622; doi:10.1186/s13023-025-03887-x)
Supplement: Supplementary file 4 — Additional file 4: Reported answers partner participants. This file includes the numbers of reported answers (raw data) on the complete survey by partner participants. [file 13023_2025_3887_MOESM4_ESM.pdf]

Additional file 4. Reported answers partner participants

Included participants	49
Age, n (%)	
Under 25 years	1 (2)
25-35 years	7 (14)
35-45 years	7 (14)
45-55 years	17 (35)
55-65 years	10 (20)
65 years and older	7 (14)
Sex, n (%)	
Female	29 (59)
Male	20 (41)
HHT type, n (%)	
HHT type 1	17 (35)
HHT type 2	16 (33)
SMAD 4	1 (2)
Unknown	13 (27)
Missing	2 (4)
VAS HHT severity, median (IQR)	6 (5.0-7.0)
Missing, n (%)	2 (4)
VAS epistaxis severity, median (IQR)	5 (3.0-7.0)
Missing, n (%)	1 (2)
Received questionnaire through:	
Patient association	16 (33)
HHT expert center	8 (16)
Newsletter	2 (4)
Social media	3 (6)
Family member	16 (33)
Other	4 (8)

Table 4. Baseline characteristics partner participants

Included participants (n)	49
Influence HHT relationships, n(%)	
Only some minor concern/ worry	11 (22)
Decided not to have a relationship	1 (2)
Decided to have a relationship	3 (6)
Decided to postpone relationship until diagnosis	0
Decided to postpone relationship until after screening/ treatment	0
There was no effect	35 (71)
Influence HHT pregnancy and children, n(%)	
Not to have children	2 (4)
To have children	4 (8)
Postpone having children	2 (4)
Have children at earlier age	1 (2)
Have fewer children	5 (10)
Have more children	0
Embryonic selection to exclude HHT	2 (4)
Adopt children	1 (2)
Perform prenatal genetic testing for HHT	3 (6)
Other	10 (20)

Not applicable	27 (55)
Reduction influence HHT on family planning*, n(%)	
Patient-friendly information	15 (31)
Answers to my questions	14 (29)
Improved access to HHT-expert center	9 (18)
Improved treatments for HHT	19 (39)
Support for other HHT patients in my family	3 (6)
Support from other HHT patients in my family	2 (4)
Patient support groups	3 (6)
Economic support	7 (14)
Other	5 (10)
I don't think anything would have helped	16 (33)

Table 5. Reported answers family planning partner participants
* multiple answers could be reported by a single patient

Included partner participants (n)	49
Sexually active, n(%)	44 (90)
Missing	0
Influence HHT on sexual activity, n(%)	
Yes	17 (35)
No	29 (59)
I don't know	2 (4)
Missing	1 (2)
Emotions in sexual life because of HHT symptoms*, n(%)	
Distress	13 (27)
Frustration	13 (27)
Sexual inadequacy	7 (14)
Dissatisfaction	6 (12)
Bothered by low sexual desire	6 (12)
Embarrassment	4 (8)
Fear of having HHT symptoms	9 (18)
Other	2 (4)
None of the above	25 (51)
VAS influence HHT complaints on sexual activity, median (IQR)	1.6 (0.2-6.1)
Missing, n (%)	1 (2)
Reduction of influence*, n(%)	
My partner is not symptomatic	5 (10)
Only mild symptoms	8 (16)
No (more) symptoms during intimacy/ sexual activity	15 (31)
My partner is very comfortable with HHT	10 (20)
I am aware of HHT and make my partner feel comfortable	25 (51)
Other	5 (10)
More information necessary, n(%)	
No, it's not necessary	10 (20)
Yes, it's necessary	21 (43)
I don't know	18 (37)

Table 6. Reported answers sexual activity partner participants
* multiple answers could be reported by a single patient

Family planning, sexual activity and contraception in hereditary hemorrhagic telangiectasia: a European survey study, Orphanet Journal of Rare Diseases, J. Hessels et al., pulmonary department St. Antonius Hospital, j.hessels@antoniusziekenhuis.nl
