# Supplementary material for: Family planning, sexual activity and contraception in hereditary hemorrhagic telangiectasia: a European survey study
Source: Orphanet J Rare Dis. 2025 Aug 1;20:395. doi: 10.1186/s13023-025-03887-x (PMC12317622; doi:10.1186/s13023-025-03887-x)
Supplement: Supplementary file 5 — Additional file 5: Subgroup analysis statistical analysis. This file includes the statistical results from the subgroup analysis (based on age and nationality) including chi-square test results, and in case of a significant association also Cramer’s V coefficient for further interpretation. [file 13023_2025_3887_MOESM5_ESM.pdf]

Additional file 5. Subgroup analysis statistical analysis

Categorical variables

Question/ answers	Association with age-subgroups: p-value (Cramer's V*)	Association with nationality-subgroups: p-value (Cramer's V*)
In what way did HHT affect your decisions concerning relationships?		
Only some minor concern and worry	0.308	<0.001 (0.263)
Decided not to have a relationship	0.427	0.044
Decided to have a relationship	0.172	0.090
Decided to postpone relationship	0.725	0.275
There was no effect	0.765	<0.001 (0.298)
In what way did HHT affect your decisions concerning pregnancy and children? HHT probably influence my/ our decision to:		
Not have children	0.001 (0.189)	0.012 (0.160)
Have children	0.002 (0.183)	0.007 (0.167)
Postpone having children	<0.001 (0.225)	0.185
Have children at earlier age	0.017 (0.155)	0.521
Have fewer children	<0.001 (0.221)	0.468
Have more children	0.776	0.149
Embryonic selection to exclude HHT	<0.001 (0.277)	<0.001 (0.205)
Adopt children	0.011 (0.161)	0.546
Perform prenatal genetic testing for HHT	<0.001 (0.245)	0.582
Other	0.856	0.974
Not applicable	<0.001 (0.291)	0.004 (0.173)
What do you think helped/ would have helped to reduce the influence of your (partners) HHT on your family planning?		
Patient-friendly information	0.022 (0.152)	0.004 (0.174)
Answers to my questions	0.003 (0.178)	0.013 (0.159)
Improved access to an HHT expert center	<0.001 (0.231)	<0.001 (0.241)
Improved treatments for HHT	0.004 (0.175)	<0.001 (0.202)
Support for other HHT patients in my family that I care for	0.213	<0.001 (0.194)
Support from other HHT patients in my family	0.15 (0.157)	0.531
Patient support groups	0.005 (0.171)	<0.001 (0.198)
Economic support	<0.001 (0.224)	0.008 (0.166)
I don't think anything would have helped me/us	0.022 (0.152)	<0.001 (0.219)
Other	0.011 (0.161)	0.026 (0.149)

In what way did HHT affect your decisions concerning pregnancy and children? HHT probably influence my/ our decision to:	Association with presence of visceral malformations (combined)	PAVM	HVM	Gastro-intestinal	CAVM
Not have children	0.280	0.892	0.284	0.312	0.071
Have children	0.336	0.561	0.963	0.602	0.873
Postpone having children	0.095	0.089	0.145	0.542	0.004 (0.120)
Have children at earlier age	0.406	0.764	0.619	0.693	0.766
Have fewer children	0.083	0.043 (0.085)	0.796	0.820	0.033
Have more children	0.238	0.840	0.813	0.556	0.156
Embryonic selection to exclude HHT	0.154	0.673	0.118	0.337	0.183

Adopt children	0.260	0.122	0.345	0.334	0.006 (0.114)
Perform prenatal genetic testing for HHT	0.937	0.346	0.911	0.437	0.013
Other	0.861	0.060	0.564	0.478	0.394
Not applicable	0.388	0.127	0.010 (0.108)	0.701	0.004

Question/ answers	Association with age-subgroups: p-value (Cramer's V*)	Association with nationality-subgroups: p-value (Cramer's V*)
Do you think having HHT has influenced your current/ potential/ previous intimacy and sexual activity?	0.230	0.005 (0.151)
Have you ever experienced the following emotions in your sexual life because of HHT symptoms?		
Distress	0.782	<0.001 (0.228)
Frustration	0.056	0.031 (0.147)
Sexual inadequacy	0.702	0.872
Dissatisfaction	0.796	0.012 (0.160)
Bothered by low sexual desire	0.819	<0.001 (0.198)
Embarrassment	0.050	0.028 (0.148)
Fear of having HHT symptoms	0.330	<0.001 (0.210)
Other	0.805	0.357
None of the above	0.853	<0.001 (0.235)
What is the consequence of these emotions?		
Low sexual desire	0.686	0.019 (0.153)
Avoid sexual activity occasionally	0.932	<0.001 (0.209)
Avoid sexual activity in general	0.772	0.571
Avoid having relationships	0.902	0.528
Other	0.017 (0.155)	0.033 (0.145)
What contributes to a reduction of the influence of your HHT on your intimacy and sexual activity?		
I am not symptomatic	0.009 (164)	<0.001 (0.306)
I only have mild symptoms	<0.001 (0.228)	0.365
I don't have (more) symptoms during intimacy or sexual activity	0.039 (0.143)	<0.001 (0.201)
I feel very comfortable with my HHT	0.198	0.349
My partner(s) have been aware of my HHT and make me feel comfortable about it	0.163	<0.001 (0.301)
Other	0.002 (0.182)	0.410
Do you think it's necessary/ important that there is more attention regarding the influence of HHT on intimacy and sexual activity?	0.697	<0.001 (0.186)

Question/ answers	Association with age-subgroups: p-value (Cramer's V*)	Association with nationality-subgroups: p-value (Cramer's V*)
Which type(s) of contraceptives did you ever use?		
I have never used contraception	0.328	0.012 (0.160)
Barrier methods	<0.001 (0.259)	0.013 (0.159)
Hormonal contraception	<0.001 (0.262)	<0.001 (0.241)
Hormonal IUD	0.002 (0.182)	0.026 (0.149)
Copper IUD	0.551	0.079
Natural contraceptives	0.001 (0.188)	0.142
Sterilization	0.598	0.042 (0.142)

Other	0.509	0.167
Do you think you were well informed about the options and their respective pros and cons before starting with contraceptives?	0.344	0.760

* *In case of a significant association, Cramer's V coefficient was used for further interpretation of the strength of the association: a value of <0,25 was considered as a weak association, 0.25-0.75 as a moderate association and >0.75 as a strong association.*

Regression analysis

Model for VAS-score for influence on sexual activity	p-value	Odds ratio	95% confidence interval for Odds ratio
Age-groups only			
Under 25 years	0.002	0.087	0.018-0.419
25-35 years	0.081	0.465	0.196-1.102
35-45 years	0.219	0.624	0.294-1.325
45-55 years	0.399	0.719	0.329-1.559
55-65 years	0.633	1.191	0.580-2.443
65 years and older	Reference cat	1	N/A
Nationalities only			
Italian	0.133	1.866	0.826-4.213
French	0.488	1.290	0.627-2.654
Danish	<0.001	0.220	0.094-0.515
German	0.635	1.248	0.499-3.121
Dutch	0.562	0.769	0.315-1.879
Other nationalities	Reference cat	1	N/A
Combined: age-groups, nationality, VAS-score epistaxis, VAS-score HHT severity			
VAS-epistaxis severity	0.003	1.191	1.062-1.336
VAS- HHT severity	<0.001	1.448	1.276-1.642
Under 25 years	0.447	0.552	0.119-2.562
25-35 years	0.823	1.096	0.491-2.447
35-45 years	0.958	1.020	0.497-2.093
45-55 years	0.623	0.834	0.404-1.723
55-65 years	0.232	1.504	0.769-2.942
65 years and older	Ref cat	1	N/A
Italian	0.114	1.864	0.859-4.044
French	0.664	1.167	0.581-2.341
Danish	0.009	0.336	0.147-0.765
German	0.903	1.055	0.444-2.506
Dutch	0.679	0.839	0.364-1.934
Other nationalities	Ref cat	1	N/A

Family planning, sexual activity and contraception in hereditary hemorrhagic telangiectasia: a European survey study, Orphanet Journal of Rare Diseases, J. Hessels et al., pulmonary department St. Antonius Hospital, j.hessels@antoniusziekenhuis.nl
